# The relationship between tetanus intensity and the magnitude of hippocampal long-term potentiation *in vivo*

**DOI:** 10.1016/j.neuroscience.2012.11.056

**Published:** 2013-02-12

**Authors:** S.J. Martin, K.L. Shires, P.A. Spooner

**Affiliations:** Centre for Cognitive and Neural Systems, The University of Edinburgh, 1 George Square, Edinburgh EH8 9JZ, Scotland, UK

**Keywords:** EPS, Evoked Potential Sampler, fEPSP, field excitatory post-synaptic potential, LTP, long-term potentiation, PPF, paired-pulse facilitation, dentate gyrus, LTP, hippocampus, synaptic plasticity, rat

## Abstract

In this study, we assessed the effects of varying tetanus and test-pulse intensity on the magnitude of long-term potentiation (LTP) in the perforant path–dentate gyrus projection of urethane-anaesthetized rats. We developed a novel within-subjects procedure in which test-pulse-stimulation intensity (60–1000 μA) was varied quasi-randomly under computer control throughout the recording period. After a baseline period, we applied a high-frequency tetanus, the intensity of which was varied over the same range as test-pulse intensity, but between subjects. The time-course of LTP was thus monitored continuously across a range of test-pulse intensities in each rat. Intense high-frequency tetanization at 1000 μA resulted in a paradoxical depression of the dentate field excitatory post-synaptic potential (fEPSP) slope at the lowest test intensity used (60 μA), but caused a potentiation at higher test intensities in the same animal. Moreover, intense tetanization induced less LTP than a moderate tetanus over most of the test-intensity range. Explanations for this pattern of data include a potentiation of feed-forward inhibition in conjunction with LTP of excitatory neurotransmission, or local tissue damage at the stimulation site. To address this issue, we conducted an additional experiment in which a second stimulating electrode was placed in the perforant path at a site closer to the dentate, in order to activate a common population of afferents at a location ‘downstream’ of the original stimulation site. After 1000-μA tetanization of the original (‘upstream’) site, fEPSPs were again depressed in response to test stimulation of the upstream site, but only potentiation was observed in response to stimulation of the downstream site. This is consistent with the idea that the depression induced by intense tetanization results from local changes at the stimulation site. In conclusion, while tetanus intensity must exceed the LTP induction threshold, intensities above 500 μA should be avoided; in the present study, tetanization at 250–500 μA yielded maximal levels of LTP.

## Highlights

► An intense tetanus causes a depression of hippocampal fEPSPs at low test intensities. ► An intense tetanus yields less LTP than a moderate tetanus at higher test intensities. ► Local changes at the stimulation site are likely to underlie these effects. ► *In vivo*, tetanus currents >500 μA should be avoided; 250–500 μA is probably optimal.

## Introduction

The induction of long-term potentiation (LTP) by the delivery of high-frequency tetanic stimulation is commonly used to assess the capacity for synaptic change in rodents following drug administration or genetic alteration (Martin et al., 2000). However, the magnitude of LTP recorded in this way is sensitive to several experimental variables that often differ between research groups and individual experiments. The stimulation current used to sample fEPSPs before and after tetanization provides one example: low-intensity test pulses typically result in a larger percentage increase in fEPSP slope than high-intensity test stimulation, a phenomenon attributed to the increasingly non-linear summation of individual post-synaptic potentials as large numbers of afferent fibres are recruited (Jeffery, 1995).

The intensity of high-frequency tetanic stimulation is another critical variable. The magnitude of LTP typically increases in parallel with tetanus intensity; as increasing numbers of afferents are activated, growing numbers of synapses reach the threshold for potentiation, a phenomenon known as cooperativity (McNaughton et al., 1978; Bliss and Collingridge, 1993). However, strong tetanization sometimes results in a paradoxical depression of the fEPSP slope at low test-pulse intensities (Hodgson et al., 1997; Trepel and Racine, 1998). This is a potential concern if, as is sometimes the case, the chosen tetanus protocol involves increasing the tetanization current substantially above the intensity used for test-pulse stimulation (e.g. Martin and Morris, 1997). There are at least two potential explanations for this tetanus-induced depression of fEPSPs: (1) a potentiation of rapid feed-forward inhibition might occur in conjunction with the enhancement of excitatory transmission (Kairiss et al., 1987); (2) strong tetanization might result in local damage to afferent fibres close to the stimulation site. For further discussion of these issues, see Trepel and Racine (1998).

In the present study, we set out to investigate this issue by recording LTP in the dentate gyrus of urethane-anaesthetized rats while systematically varying both tetanus and test-pulse intensity. To achieve this, we used a novel within-subjects stimulation protocol in which test-pulse intensity was varied quasi-randomly during both pre- and post-tetanus recording sessions—in other words, baseline stimulation comprised a continuous input/output curve. Tetanus intensity was varied between subjects. An additional, two-pathway experiment was subsequently carried out in order to distinguish between the possibilities outlined above.

## Experimental procedures

### Subjects

All procedures were conducted in accordance with the UK Animals (Scientific Procedures) Act (1986), and subjected to local ethical review at the University of Edinburgh. Prior to the experiment, young adult male Lister-hooded rats, 250–500 g in weight, and ranging in age from approximately 10 to 20 weeks (*n* = 48) were given *ad libitum* access to food and water and maintained on a 12-h light/12-h dark cycle. All rats were obtained from a commercial supplier (Charles River, UK), and had previously undergone purely behavioural testing, without drug administration or any other intervention, in unrelated watermaze experiments. Without existing data on the effects of systematic changes in tetanus and test intensity of the variability in LTP, we were unable to conduct formal power analyses in advance in order to determine appropriate sample sizes. However, based on previous studies using standard parameters, we estimated that an *n* of 6 was the smallest likely to yield robust data; accordingly, this sample size was used throughout experiments 1 and 2.

### Electrophysiology

Rats were anaesthetized with urethane (ethyl carbamate; 1.5 g/kg; 0.3 mg/ml, i.p.) and placed in a stereotaxic frame (Kopf, Tujunga, CA, USA) with the skull horizontal. Body temperature was monitored by a rectal probe and maintained at 36.2 °C using an isothermic heating blanket. Depth of anaesthesia was assessed throughout the experiment, and urethane top-ups of 0.2 ml were administered as required. A polytetrafluoroethylene-insulated monopolar platinum/iridium recording electrode (outer diameter = 0.103 mm) was lowered unilaterally into the hilus of the dentate gyrus (AP = −3.5 mm from bregma; ML = 2.0 mm; DV (from dura) = ca. −3.0 mm), and a bipolar stimulating electrode comprising two twisted wires identical in composition to the recording electrode was lowered into the angular bundle of the perforant path (AP = −7.5 mm; ML = 4.0 mm; DV (from dura) = ca. −2.5 mm). fEPSPs were amplified, band-pass filtered between 1.0 Hz and 5 kHz, and sampled at 20 kHz using a PC running custom-written LabView software—Evoked Potential Sampler (EPS) developed by Patrick Spooner. fEPSP slope (measured by linear regression between two fixed time points) was monitored on-line. Stimulation was delivered under computer control via a Neurolog system (NL800A; Digitimer Ltd., Herts., UK), and consisted of biphasic constant-current pulses. All stimulation parameters, including timing, pulse duration, and current, were controlled by a computer based on information provided before the start of recording. Correct placement of electrodes was determined on the basis of characteristic changes in fEPSP shape and amplitude during implantation, particularly the negative-to-positive reversal observed upon moving the recording electrode from the molecular layer to the granule-cell layer of the dentate gyrus. Final electrode depths were adjusted to maximize the amplitude of the positive-going dentate fEPSP elicited by perforant path stimulation. After electrode implantation, stimulation was turned off for 30 min to allow the brain tissue to settle, after which baseline recording was started.

### Experiment 1

Single biphasic test pulses (100 μs per phase) were delivered to the stimulating electrode at 20-s intervals. The current used for test-pulse stimulation (referred to throughout as the ‘test intensity’) was selected randomly by our EPS software, and varied between 60, 120, 250, 500, and 1000 μA, subject to the constraint that all five intensities were sampled once in each successive block of five test pulses. In this way, baseline stimulation comprised a continuous input/output curve conducted in a random sequence in order to balance, over time, any potential hysteresis effects caused by strong test stimulation followed by weak stimulation and *vice versa*. Once a stable baseline recording had been obtained for at least 1 h, a high-frequency tetanus was delivered. Tetanization consisted of three trains of 50 pulses at 250 Hz, with a 60-s inter-train interval. Tetanus intensities varied between subjects over the following range: 0 (no tetanus), 60, 120, 250, 500, and 1000 μA; *n* = 6 in all cases. The sequence of tetanus intensities delivered in successive experiments was randomly generated by a computer in exactly the same way that test intensities were chosen in a within-subjects manner. After tetanization, baseline stimulation resumed for a 1-h period. For each combination of baseline stimulation intensity and tetanus intensity, fEPSP slope data were normalized to the mean of the 1-h pre-tetanus period (assigned a value of 100%), and group means were calculated. Data from all rats in which recordings were made are included in the final analysis; no data were excluded.

### Experiment 2

In experiment 2, a second stimulating electrode (AP = −6.5; ML = 3.0; DV = ca. −2.5 mm from dura) was added anterior to the first, in order to stimulate a common population of perforant path afferents at a point downstream of the first electrode. These two stimulation sites are therefore termed ‘upstream’ and ‘downstream’. All procedures were identical to those described for experiment 1—including the randomization of stimulation intensity—except that stimulation alternated between each stimulation site every 20 s. High-frequency tetanization was delivered to the upstream site only, always at an intensity of 1000 μA (*n* = 6). In order to compare the effects of a high-intensity tetanus with those of a small marking lesion, an additional group (*n* = 6) received a 1000-μA biphasic constant-current pulse, 1 s per phase, in place of tetanic stimulation.

In order to confirm that both upstream and downstream stimulation sites recruit overlapping populations of perforant path fibres even at very low stimulation intensities, paired-pulse stimulation was carried out in two of the animals before the start of the main experiment. During this phase, the recording electrode was placed in the molecular layer of the dentate gyrus in order to record negative-going dendritic fEPSPs. Pairs of biphasic stimulation pulses (100 μs per phase) were delivered at 60 μA, comprising initial stimulation of the downstream site followed, at an interval of 50 ms, by stimulation of the upstream site (six pairs; 10-s intra-pair interval). This was followed by a series of single stimulation pulses delivered to the downstream site only (six pulses; 10-s intra-pulse interval). Paired-pulse facilitation (PPF) was calculated by expressing the mean fEPSP slope recorded in the upstream pathway after stimulation of the downstream site as a percentage of the value obtained in the upstream pathway without prior stimulation.

### Histology

At the end of each experiment, rats were killed by cervical dislocation and brains were removed and stored in 10% formalin. Thirty-micrometre coronal sections through the hippocampus were then cut using a cryostat: one in three sections was mounted on a slide, stained with Cresyl Violet, and examined under a light microscope. All electrodes were correctly located.

## Results

**Fig. 1:**
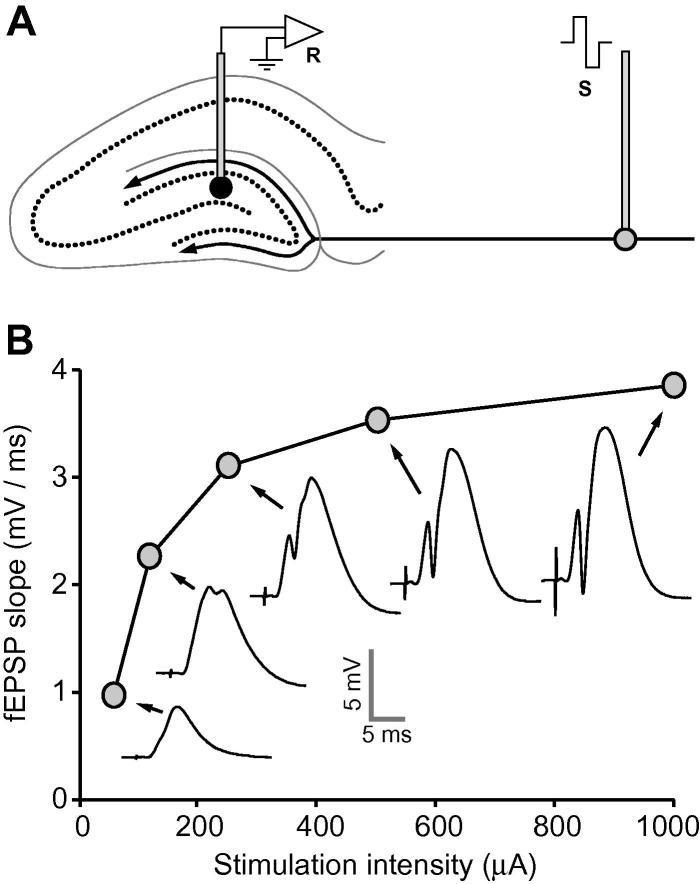
(A) Schematic diagram of the placement of electrodes in experiment 1—a stimulating electrode (S) in the perforant path and a recording electrode (R) in the hilus of the dentate gyrus. (B) Relationship between fEPSP and test-pulse stimulation intensity in a single rat. Examples of fEPSPs elicited by stimulation at each of the intensities sampled are shown.

Stimulation of the perforant path (Fig. 1A) resulted in positive-going fEPSPs in the hilus of the dentate gyrus. Fig. 1B shows the rise in fEPSP slope with increasing stimulation intensity in a single rat; representative fEPSPs are shown at each intensity. Data were collected from a single animal during the pre-tetanus baseline period (throughout which stimulation intensity was varied quasi-randomly), and mean values were then calculated. Note the appearance of a population spike at intensities of 120–250 μA and above.

**Fig. 2:**
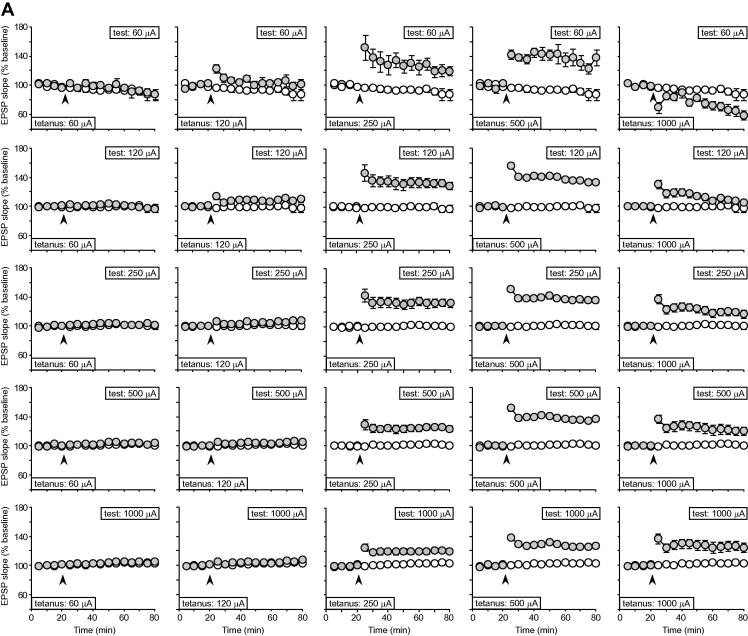
The time course of LTP across the full range of tetanus intensities (columns), and test intensities (rows). All data are normalized to the mean value obtained over the 20-min baseline period. In each panel, data from the non-tetanized control group at the corresponding test intensity (white circles) are plotted alongside the data from the relevant tetanized group (grey circles) for comparison.

**Fig. 3:**
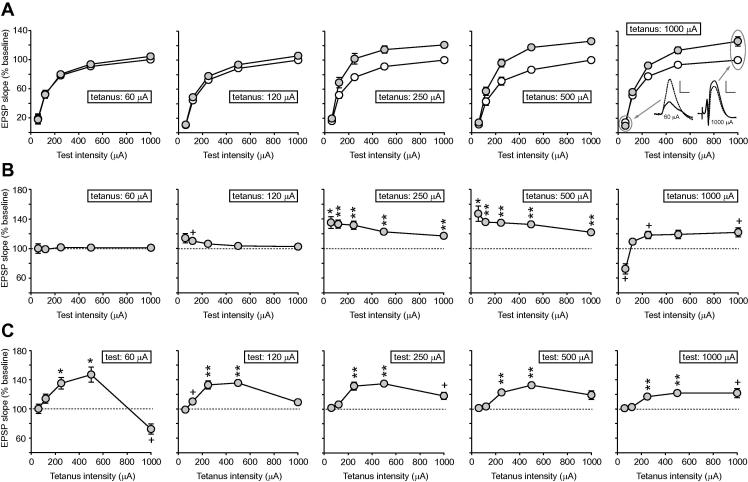
(A) Input/output curves relating fEPSP slope to test intensity both before and after tetanization. Data from each rat were normalized to the mean value obtained following test stimulation at 1000 μA before tetanization (arbitrarily designated 100%), and group means were calculated. In the top-right panel, examples of fEPSPs recorded before (dotted line) and after tetanization at 1000 μA (solid line) are shown at test intensities of 60 μA (left-hand side; scale bar = 1 mV (vertical) and 5 ms (horizontal)) and 1000 μA (right-hand side; scale bar = 5 mV (vertical) and 5 ms (horizontal)). (B) Relationship between mean LTP recorded 40–60 min after tetanization and test intensity across the range of tetanus intensities studied. (C) Relationship between LTP and tetanus intensity across the range of test intensities sampled. In both B and C, the significance level of potentiation or depression at each point, relative to baseline (100%; dotted line), is indicated on the graphs: ^+^0.1 > *p* > 0.05; ^∗^*p* < 0.05; ^∗∗^*p* < 0.01.

Fig. 2 shows the time course of LTP as a function of both tetanus intensity (left to right; between subjects), and stimulation intensity (top to bottom; within subjects), for experiment 1. Each panel represents the effect of a specific tetanus intensity on fEPSP slope potentiation at a single test intensity. The threshold for LTP induction was reached at tetanus intensities of 120–250 μA. For example, at a test intensity of 120 μA, two out of six rats exhibited >10% LTP after 1 h, but at a tetanus intensity of 250 μA, all six rats showed >10% potentiation. In general, LTP reached its highest values at tetanus intensities of 250–500 μA, but was reduced at the highest intensity of 1000 μA. In fact, tetanization at 1000 μA caused a depression, rather than a potentiation, of fEPSP slope at the lowest test intensity sampled, 60 μA (top-right panel). As expected, however, LTP at moderate tetanus intensities tended to be largest at lower test intensities, although this effect was masked somewhat by the slight baseline fall observed, particularly at a test intensity of 60 μA. In the absence of tetanization (first column), low test intensities were associated with a gradual fall in fEPSP slope, whereas a slight rise was evident at high stimulation intensities; little change was observed at moderate intensities. An ANOVA of normalized fEPSP slope in this group, in which both test intensity and time were entered as within-subject factors, revealed a significant interaction of test intensity and time [*F*(60,300) = 2.24; *p* < 0.001], and there was a significant rise in mean fEPSP slope over the final 20 min of recording as test intensity increased [*F*(4,20) = 5.17; *p* < 0.01]. A statistical analysis of the LTP data is presented in connection with Fig. 3. No differences in absolute baseline fEPSP slope values were observed between the different tetanus-intensity groups [Table 1; *F* < 1], and there were no mean differences in weight (a proxy for age) between the tetanus-intensity groups [0 μA = 339.2 ± 36.8 g; 60 μA = 335.8 ± 28.6 g; 120 μA = 333.5 ± 24.3 g; 250 μA = 321.8 ± 31.3 g; 500 μA = 340.2 g; 1000 μA = 348.3 ± 31.7 g; *F* < 1].

Fig. 3A shows input/output curves comparing mean fEPSP slope values recorded over the 20-min baseline period, and 40–60 min after tetanization, across the range of tetanus intensities (left to right). For each rat, fEPSP slope data were normalized to the value elicited by 1000-μA stimulation prior to tetanization, and group means were calculated. Note the fall in fEPSPs elicited by low test intensities, and the potentiation of fEPSPs elicited by high test intensities after 1000-μA tetanization. However, in order to examine the magnitude of LTP across the input/output curve, it is more informative to plot post-tetanus fEPSP values as a percentage of baseline values. Accordingly, we calculated the percentage LTP obtained 40–60 min after tetanization for all combinations of tetanus and test intensity. In order to remove the confounding effects of the differential baseline changes observed at different test intensities, we normalized data in all cases to the value obtained in the non-tetanized group at the corresponding test intensity. Fig. 3B shows the relationship between test intensity and LTP across the range of tetanus intensities. As expected, at tetanus intensities above the threshold for LTP induction, and after normalizing to remove the effect of baseline drift, potentiation was greatest at low test intensities—except at the very highest tetanus intensity of 1000 μA. An overall ANOVA of normalized fEPSP slope 40–60 min after tetanization revealed a significant interaction of tetanus and test intensity [*F*(16,100) = 9.04; *p* < 0.001]. Potentiation decreased significantly with increasing test intensity at tetanus intensities of 250 μA [*F*(4,20) = 7.67; *p* < 0.005] and 500 μA [*F*(4,20) = 3.41; *p* < 0.05]. However, potentiation *increased* significantly with rising test-stimulus intensity after tetanization at 1000 μA [*F*(4,20) = 15.5; *p* < 0.001]. Following the ANOVAs outlined above, a series of one-sample *t*-tests (comparison to 100%, i.e. baseline) with Bonferroni corrections established the significance level of each combination of tetanus and test intensity; these values are indicated in Fig. 3B, C. In Fig. 3C, the same data are re-plotted to examine the relationship between tetanus intensity and LTP across the range of test intensities. This function has an inverted-U shape across most of the range, with maximal potentiation at test intensities of 250–500 μA. This shape was especially pronounced at the lowest test intensity of 60 μA, at which a 1000-μA tetanus caused a depression of fEPSP slope. The overall effect of tetanus intensity was highly significant [*F*(4,25) = 21.2; *p* < 0.001], and individual ANOVAs revealed significant effects of tetanus intensity at each test intensity [*F*(4,25) > 9.0 in all cases; *p* < 0.001]. Analysis of the fall in potentiation between tetanus intensities of 500 and 1000 μA revealed a significant drop at test intensities of 60 μA [*t*(10) = 5.95; *p* < 0.001] and 120 μA [*t*(10) = 5.14; *p* < 0.001], and a non-significant trend at 250 μA [*t*(10) = 2.88; 0.1 > *p* > 0.05; independent samples *t*-tests with Bonferroni correction].

**Fig. 4:**
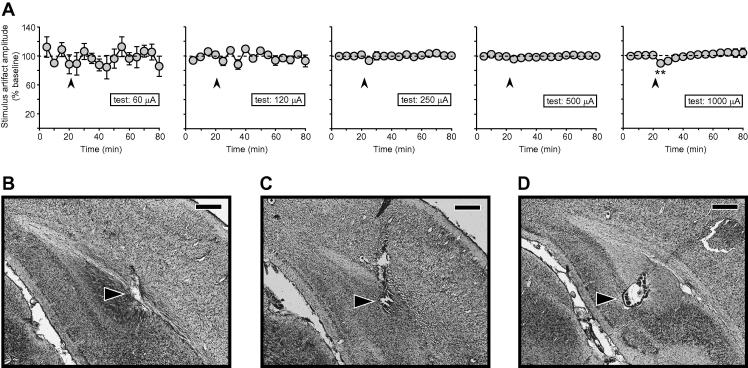
(A) The amplitude of the stimulus artefact (mean of positive and negative components) is plotted as a function of test-pulse intensity and time for the 1000-μA-tetanus group only, and normalized to the pre-tetanus value at each intensity. The stimulus artefact remained stable over time and, at low test intensities, was unaffected by tetanization. A small, transient decrease in the size of the artefact was observed at high test intensities (^∗∗^*p* < 0.01; post hoc one-sample *t*-tests with Bonferroni correction). (B–D) Photomicrographs of the stimulation site (black arrows) in Cresyl-Violet-stained brain sections. Representative examples are shown from animals that received (B) a 1000-μA tetanus, (C) no tetanus, and (D) a small marking lesion. Scale bar = 0.5 mm.

To examine the possibility of local changes to the passive electrical properties of the brain tissue surrounding the stimulating electrode, or the electrode itself, we measured the amplitude of the stimulus artefact (mean of the positive- and negative-going components) as a function of test intensity, tetanus intensity, and time. Fig. 4A shows the amplitude of the stimulus artefact across the duration of the experiment in the 1000-μA-tetanus group; data are plotted separately for each test intensity, and normalized to the mean of the 20-min pre-tetanus value. There was no systematic drift in the size of the stimulus artefact over time at any test intensity, but tetanization resulted in a small reduction in the stimulus artefact over the 5-min period immediately after the tetanus at a test intensity of 1000 μA [*t*(5) = 6.17; *p* < 0.01; Fig. 4A; far-right panel], an effect that no longer reached significance during the next 5-min block; no significant changes were observed at other test intensities (post-hoc one-sample *t*-tests with Bonferroni correction). The absolute amplitude of the stimulus artefact increased with test-pulse current as follows—60 μA: 0.04 ± 0.01 mV; 120 μA: 0.12 ± 0.03 mV; 250 μA: 0.33 ± 0.11 mV; 500 μA: 0.70 ± 0.24 mV; 1000 μA: 1.17 ± 0.33 mV. Tetanization at lower intensities had no effect on the stimulus artefact, even at the highest test intensity (data not shown).

Inspection of Cresyl-Violet-stained coronal sections did not reveal any visible tissue damage at the stimulation site, even following tetanization at 1000 μA, relative to non-tetanized controls. Fig. 4B shows an example of the stimulation site following 1000 μA stimulation, compared to that in a non-tetanized animal (Fig. 4C). Fig. 4D shows a small marking lesion for comparison (see experiment 2 below).

**Fig. 5:**
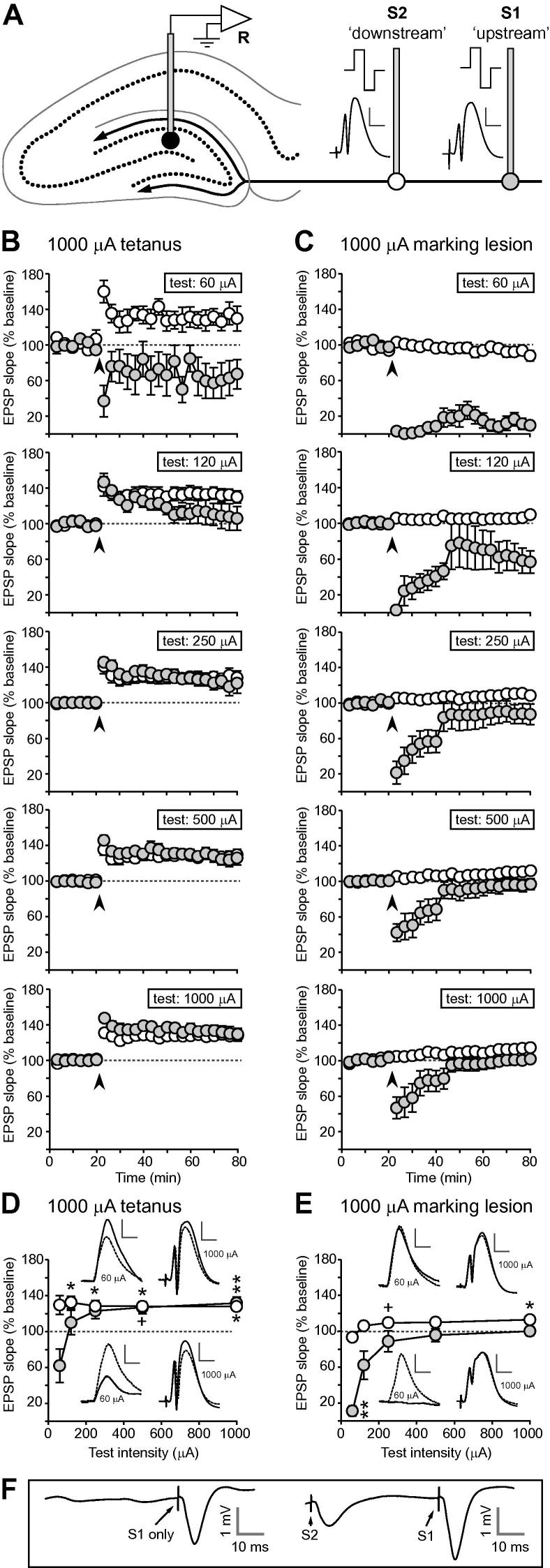
Experiment 2. (A) Schematic diagram of the placement of upstream (S1; white circle at tip) and downstream (S2; grey circle at tip) stimulating electrodes within the perforant path in experiment 2. Typical examples of fEPSPs elicited by 1000-μA test stimulation at both sites are shown. (B) The time course of LTP at each test intensity elicited by a 1000-μA upstream tetanus. Responses to stimulation of both upstream (filled circles) and downstream (open circles) pathways are shown; dotted lines indicate baseline (i.e. 100%). (C) The effects of a small marking lesion delivered via the upstream stimulating electrode on fEPSP slope in upstream (filled circles) and downstream (white circles) pathways at each test intensity. (D) Relationship between normalized fEPSP slope and test intensity in both upstream and downstream pathways after 1000 μA tetanization. Examples of fEPSPs recorded in both pathways are shown (below graph = upstream pathway; above graph = downstream pathway) before (dotted lines) and after tetanization of the upstream site (solid lines), in response to stimulation at 60 μA (left-hand side; scale bar = 1 mV (vertical) and 5 ms (horizontal)) and 1000 μA (right-hand side; scale bar = 5 mV (vertical) and 5 ms (horizontal)). (E) Relationship between normalized fEPSP slope and test intensity in both upstream and downstream pathways after marking stimulation. Examples of fEPSPs recorded in both pathways before and after marking stimulation are shown (details as in D). In both D and E, the significance level of potentiation or depression at each point, relative to baseline (100%; dotted line), is indicated on the graphs: ^+^0.1 > *p* > 0.05; ^∗^*p* < 0.05; ^∗∗^*p* < 0.01. Symbols above the graph refer to the downstream pathway, and symbols below the graph refer to the upstream pathway. (F) Example of PPF following successive stimulation of S2 followed by S1 at an interval of 50 ms. fEPSPs were recorded from the dentate molecular layer. Stimulation of S1 alone resulted in a mean fEPSP slope of −0.46 mV/ms; prior stimulation of S2 resulted in a mean slope of −0.62 mV/ms in response to subsequent stimulation of S1, an increase of 35.1%.

In experiment 2, stimulating electrodes were placed at ‘upstream’ and ‘downstream’ locations within the ipsilateral perforant path; aside from a small difference in onset latency, both stimulation sites yielded similar fEPSPs (Fig. 5A). Fig. 5B shows the time course of LTP at each test intensity, for both upstream and downstream stimulation sites, following tetanization at 1000 μA. A depression of fEPSP slope at low test intensities was seen only after stimulation of the upstream, tetanized, location. For comparison, the effects of a small marking lesion (see Fig. 4D) are plotted in Fig. 5C. Note that both tetanization and marking stimulation result in a fall in fEPSP slope at the lowest test intensities in response to stimulation of the upstream (i.e. tetanized or lesioned) site. No depression was seen in the downstream pathway—after tetanization, this pathway was potentiated at all test intensities, and marking stimulation at the upstream site had little effect. No differences in absolute baseline fEPSP slope were observed between upstream and downstream stimulation sites [*F* < 1], and no difference was observed between the 1000-μA-tetanus and marking-lesion groups [*F* < 1; Table 2]. There was no mean group difference in weight (a proxy for age) between the two groups [tetanus = 325.8 ± 10.1 g; marking lesion = 310.0 ± 15.3 g; *F* < 1].

Fig. 5D, E summarizes the change in fEPSP slope 40–60 min after 1000-μA tetanization or marking stimulation, respectively, normalized to baseline values. Unlike the data in Fig. 3B, C, values were not normalized to control for baseline drift, as a non-tetanized control group was not included in experiment 2. Note the similar shape of the curves relating fEPSP slope changes to test intensity in both tetanus and marking-stimulation groups, despite a *y*-shift of approximately 40 percentage points between the two groups. Examples of fEPSPs recorded before and after tetanization and marking stimulation, in response to test stimulation of 60 and 1000 μA, are shown in Fig. 5D, E. Whereas strong tetanization reduced the size of the 60-μA fEPSP (Fig. 5D), in the example shown, responses were completely abolished at this test intensity after marking stimulation (Fig. 5E). In the downstream pathways, no depression was observed at low test intensities except for the small change resulting from baseline drift (see Fig. 5C, top panel), and the overall difference of 20–40 percentage points reflects potentiation following strong tetanization, but not marking stimulation. An ANOVA of normalized fEPSP slope 40–60 min after 1000-μA tetanization revealed a significant interaction of pathway (upstream versus downstream) and test intensity [*F*(4,40) = 15.4; *p* < 0.001]. In the upstream pathway, fEPSP slope fell with decreasing test intensity [*F*(4,20) = 21.4; *p* < 0.001], whereas test intensity had no effect in the downstream pathway [*F* < 1]. There was a significant difference between upstream and downstream pathways at a test intensity of 60 μA [*t*(10) = 3.16; *p* < 0.05; one-sample *t*-test with Bonferroni correction]. An ANOVA of normalized fEPSP slope 40–60 min after marking stimulation also revealed a significant interaction of pathway and test intensity [*F*(4,40) = 19.7; *p* < 0.001], and a significant overall difference between pathways [*F*(1,10) = 16.4; *p* < 0.005]. fEPSP slope increased with rising test intensity in both upstream [*F*(4,20) = 33.0; *p* < 0.001] and downstream pathways [*F*(4,20) = 26.3; *p* < 0.001]; the latter result is explained by the slight baseline fall evident at low test intensities together with a rise at high test intensities (see Fig. 5C), a phenomenon that was also evident in the non-tetanized group of experiment 1. Following the ANOVAs outlined above, a series of one-sample *t*-tests (comparison to 100%, i.e. baseline) with Bonferroni corrections established the significance level of potentiation at each test intensity in both tetanus and marking-lesion groups, and both upstream and downstream pathways (see Fig. 5B, C).

In order to confirm that both upstream and downstream stimulation sites recruited common populations of afferent fibres even at the lowest test intensities, we examined PPF between the two pathways while recording negative-going fEPSPs in the molecular layer of the dentate gyrus (see Bliss et al., 2007) in two animals, before the start of the main experiment. Paired-pulse stimulation of upstream followed by downstream sites at an interval of 50 ms revealed pronounced PPF in both cases tested. An example is shown in Fig. 5F: fEPSPs elicited by stimulation of the upstream electrode are shown with and without prior stimulation of the downstream site.

## Discussion

The results of experiment 1 confirm that, within the same animal, a strong tetanus produces a depression of the dentate fEPSP elicited by weak test stimulation, whereas a potentiation of the response is observed at higher test intensities. This finding is consistent with previous reports of similar phenomena in the dentate gyrus and neocortex (Hodgson et al., 1997; Trepel and Racine, 1998), and preliminary evidence from our own laboratory reveals a similar pattern of results when CA1 LTP is assessed following potentiation of the Schaffer-collateral input by ipsilateral tetanization of CA3 (Stephen Martin, unpublished observations). No visible tissue damage was evident at the perforant-path stimulation site in the present study, and tetanization at the highest intensity of 1000-μA caused no changes in the amplitude of the stimulus artefact at low test intensities, suggesting that changes to the electrode properties or the local resistance of the tissue cannot explain the pattern of results we have obtained. However, it is possible that very strong tetanization causes subtle pathological changes that impair the recruitment of afferent axons at locations very close to the stimulation site. Assuming that increasing tetanization currents recruit axons within an expanding area of the perforant path (see Ranck, 1975, for discussion), this would explain the observation of depression at low test intensities when only those axons passing very close to the tip of the stimulating electrode are activated.

However, a potentiation of feed-forward inhibition might also account for this pattern of results. To rule out this possibility, we examined the effects of intense tetanization on the potentiation recorded in response to stimulation of a downstream site within the same ipsilateral perforant path. No depression was observed in the downstream pathway; instead, potentiation was observed even at the lowest test intensities. This result cannot be attributed to the sampling of independent populations of afferents at the two stimulation sites, a possible concern at very low test intensities, because pronounced PPF was evident when stimulation was delivered alternately to upstream and downstream sites at an interval of 50 ms, and at the lowest test intensity of 60 μA. This result confirms that both stimulation sites recruit common, or at least overlapping, populations of afferent fibres. Overall, this pattern of results is consistent with the idea that a local deleterious effect of intense tetanization is to blame for the fEPSP depression seen after intense tetanization. In fact, a small marking lesion made via the stimulating electrode resulted in a similar, but more pronounced, depression of fEPSPs elicited by low-intensity test stimulation. It is unclear from our data whether the depressive effects of strong tetanization are transient or permanent; however, the preliminary findings of Hodgson et al. (1997) suggest the former possibility.

Aside from the maximum tetanus intensity of 1000 μA, increasing tetanus strength resulted in greater LTP, beyond an induction threshold of 120–250 μA. This was generally true, regardless of the test intensity used to sample the potentiation. As reported previously, the percentage LTP recorded typically becomes smaller at higher test intensities. This is to be expected at test intensities greater than the tetanus intensity, as test stimulation may activate afferents not recruited during tetanization. However the same relationship was also observed at test intensities below the tetanus intensity. This phenomenon has been attributed to the increasingly linear summation of individual post-synaptic potentials when only a small number of afferent fibres are activated, and may explain the relatively larger percentage potentiation observed in hippocampal slice experiments, in which the number of afferent fibres activated is likely to be smaller than the number recruited by stimulation in the intact animal (Jeffery, 1995). However, in the present experiment, the advantages of low test intensities were offset by a downward baseline drift at the lowest intensities; for this reason, very low test intensities are probably best avoided in practice.

Despite the downward drift in fEPSP slope observed at low test intensities, high test intensities typically yielded a rising baseline in the same animal. This phenomenon has been reported previously, and attributed to a gradual recovery from the mechanical trauma associated with electrode implantation (Gilbert and Mack, 1999). However, Gilbert and Mack (1999) report similar effects even at low test intensities, although they do observe a gradual fall in baseline in urethane-injected animals with chronically implanted electrodes (see also Riedel et al., 1994). The reasons for these differences are unclear, but it is possible that the pattern of data obtained in the present study reflects a combination of recovery from local tissue trauma superimposed on the effects of urethane anaesthesia. Regarding our use of anaesthesia, it is worth noting that urethane suppresses fEPSPs and necessitates the use of stronger tetanization parameters for LTP induction (Riedel et al., 1994; Gilbert and Mack, 1999; see Albensi et al., 2007). However, urethane is a commonly used anaesthetic for rodent LTP experiments in our own laboratory as well as others, and we were specifically interested in determining the effects of tetanus and test-pulse intensity under these conditions. Nonetheless, Hodgson et al. (1997), using freely moving rats, have reported evidence for a similar pattern of potentiation and depression to that presented here, suggesting that the phenomenon is not simply an artefact of anaesthesia.

Although not addressed in the experiments presented here, the pattern of tetanic stimulation employed is also a critical factor in determining the magnitude and persistence of LTP. For example, theta-patterned stimulation is as effective, or more effective, than conventional trains of high-frequency stimulation (Larson et al., 1986; Greenstein et al., 1988; see Albensi et al., 2007 for review). However, in this study we were specifically interested in assessing the effects of a ‘traditional’ tetanus comprising simple trains of high-frequency stimulation, a type of stimulation that is still widely used. Tetanus frequencies used to induce dentate LTP *in vivo* tend to be higher than those that are effective in CA1 *in vitro* (Albensi et al., 2007; Bowden et al., 2012), with 250 and 400 Hz being typical values (see Steward et al., 2007 for a discussion of the differences in the LTP induced using these two frequencies). We chose the former frequency because we have used 250-Hz tetanization in several previous studies, and were interested in identifying optimal parameters for LTP induction under similar conditions. In fact, a tetanus very similar, or identical, to the one used in the present study has been used in many studies of *in vivo* LTP (e.g. Martin, 1998; Vereker et al., 2000; Rosenblum et al., 2002; Bast et al., 2005; Shires et al., 2012).

In order to minimize the overall number of animals undergoing experimental procedures, we used rats that had previously undergone purely behavioural testing in a watermaze task as subjects. Although we cannot definitively exclude the possibility that this prior experience might have affected the level of potentiation observed, we have not previously observed systematic differences in the levels of LTP obtained in rats with prior watermaze experience versus experimentally naïve animals. For example, in a study using an identical tetanus protocol in naïve rats, and with tetanus and test-pulse intensities typically ranging between 200 and 300 μA (Martin, 1998; Fig. 2B), we obtained a level of LTP nearly identical to that observed using tetanus and test-pulse intensities of 250 μA in the present study (Fig. 2). Moreover, prior experience was equivalent across tetanus groups, and cannot account for the differential effects of strong versus weak tetanization.

In summary, intense tetanization can result in sub-optimal levels of LTP, or even depression, and tetanus intensities above 500 μA should probably be avoided in preparations similar to that used in the present study. Similarly, moderate test-pulse intensities (in our hands, 120–500 μA; and equal to or slightly lower than the tetanus intensity) are desirable in order to balance the advantage of increased LTP at low test intensities against the disadvantage of a falling baseline.

## Figures and Tables

**Table 1:** Baseline fEPSP slope in experiment 1. Mean baseline fEPSP slope is presented as a function of tetanus intensity (between subjects) and test intensity (within subjects)

fEPSP slope (mV/ms)	Tetanus intensity (μA)					
	0	60	120	250	500	1000
*Test intensity (μA)*						
60	0.74 ± 0.13	0.80 ± 0.25	0.60 ± 0.16	0.64 ± 0.10	0.53 ± 0.11	0.66 ± 0.11
120	2.24 ± 0.38	2.42 ± 0.23	2.39 ± 0.28	2.19 ± 0.31	2.08 ± 0.27	2.34 ± 0.28
250	3.53 ± 0.50	3.63 ± 0.32	3.92 ± 0.45	3.26 ± 0.46	3.45 ± 0.31	3.52 ± 0.35
500	4.19 ± 0.56	4.24 ± 0.40	4.76 ± 0.47	3.88 ± 0.51	4.28 ± 0.43	4.32 ± 0.41
1000	4.65 ± 0.59	4.70 ± 0.49	5.34 ± 0.49	4.30 ± 0.59	4.96 ± 4.96	4.55 ± 0.48

**Table 2:** Baseline fEPSP slope in experiment 2. Mean baseline fEPSP slope is shown as a function of test intensity in both ‘upstream’ and ‘downstream’ pathways, and tetanus and marking-lesion groups

fEPSP slope (mV/ms)	Tetanus		Marking lesion	
	Upstream	Downstream	Upstream	Downstream
*Test intensity (μA)*				
60	0.47 ± 0.11	0.65 ± 0.16	0.57 ± 0.09	0.82 ± 0.15
120	2.35 ± 0.42	2.52 ± 0.43	2.16 ± 0.16	2.65 ± 0.31
250	3.74 ± 0.53	4.04 ± 0.51	3.76 ± 0.21	3.90 ± 0.43
500	4.56 ± 0.53	5.17 ± 0.53	4.76 ± 0.37	4.94 ± 0.62
1000	5.00 ± 0.51	6.10 ± 0.62	5.33 ± 0.42	5.62 ± 0.66
